# *ASXL1* frameshift mutations drive inferior outcomes in CMML without negative impact in MDS

**DOI:** 10.1038/s41408-017-0004-0

**Published:** 2017-11-27

**Authors:** David A Sallman, Rami Komrokji, Thomas Cluzeau, Christine Vaupel, Najla H Al Ali, Jeffrey Lancet, Jeff Hall, Alan List, Eric Padron, Jinming Song

**Affiliations:** 1 Malignant Hematology Department, H. Lee Moffitt Cancer Center and Research Institute, Tampa, FL 36612 USA; 2 Cote D’azur University, Nice Sophia Antipolis University, Hematology Department, CHU Nice, Nice, 06200 France; 3 INSERM U1065, Mediterranean center of molecular medicine, Nice, 06200 France; 4 Navigate BioPharma Services, Inc., a Novartis company, Carlsbad, CA 92008 USA; 5 Hematopathology and Laboratory Medicine Department, H. Lee Moffitt Cancer Center and Research Institute, Tampa, FL 36612 USA

Next-generation sequencing (NGS) has revolutionized the diagnostic, prognostic and treatment paradigms in myeloid malignancies. Although somatic mutations can be identified in the majority of patients with myelodysplastic syndromes (MDS) and chronic myelomonocytic leukemia (CMML), the mutational spectrum and prognostic significance of individual mutations is dependent on disease subtype and remains incompletely understood^1,2^. In CMML, mutations in the addition of sex combs-like 1 (*ASXL1*) gene are the only mutations consistently associated with inferior survival in multivariable analysis and as such refine discrimination of clinical prognostic models^3,4^. Nonetheless, these studies also suggest that the type of mutation may have prognostic significance as *ASXL1* missense mutations are not similarly predictive of clinical outcomes^5^. Additional studies have confirmed the prognostic significance of *ASXL1* mutations in CMML while also identifying mutations of *CBL*, *RUNX1*, *NRAS*, and *SETBP1* to have prognostic relevance^6^. In MDS, mutations involving *TP53*, *EZH2*, *ETV6*, *RUNX1*, and *ASXL1* genes are associated with inferior survival, albeit *ASXL1* mutations are of only borderline significance^7,8^. Importantly, the prognostic relevance of type of *ASXL1* mutation has not been analyzed in MDS patients. These data suggest that there are distinct disease-specific implications in MDS versus CMML that apply to a similar mutational spectrum. Given the predicted differences in prognostic significance of *ASXL1* between MDS and CMML, we sought to compare the impact of *ASXL1* mutations in a combined cohort comprised of both diseases. We identified that although *ASXL1* mutation was predictive for outcome in CMML (HR 2.97, 95% CI 1.21–7.06; *P* = 0.02), it had no impact on outcome in MDS patients (HR 1.04, *P* = 0.87). In addition, whereas the negative significance of *ASXL1* mutation status was dependent on frameshift (FS) mutations (HR 3.85, 95% CI 1.84–15.61, *P* = 0.0026) in CMML, the type of mutation had no impact on MDS prognosis even when accounting for missense mutations.

Patients were identified retrospectively from the Moffitt Cancer Center (MCC) database that had NGS performed and a diagnosis of CMML or MDS according to WHO criteria (including patients with refractory anemia with excess blasts in transformation according to FAB). Pathology review was performed at MCC. This study was approved by the MCC Scientific Review Committee and institutional review board. From May 2013 to July 2015, a total of 60 CMML patients were identified who underwent NGS who were compared to a cohort of 195 MDS patients (Table 1). From May 2013 to October 2014, targeted amplicon based NGS of 21 myeloid genes was performed on DNA extracted from mononuclear cells of BM aspirate or peripheral blood as previously described, which was followed by NGS using a 32 gene panel^9^. The lower limit of detection for variant calling was set at a 5% variant allele frequency (VAF) and the minimum depth of coverage was 500×. Clinical characteristics were cataloged from the date of mutation analysis. Kaplan–Meier estimates were used to estimate OS and analyzed from the date of mutation identification. Cox regression models were cr7eated to adjust for clinical characteristics. Categorical and continuous variables were compared by Fisher’s exact test and Mann–Whitney’s test, respectively. All tests were two sided with statistically significant variables set at *P* < .05.

**Table 1:** Baseline characteristics of the study population by ASXL1 mutation status (frameshift and nonsense)

	**MDS cohort**		**CMML cohort**	
	*ASXL1* MT	*ASXL1* WT	*ASXL1* MT	*ASXL1* WT
	*n* = 36	*n* = 159	*n* = 28	*n* = 32
Median age (years)	74 (51–92)	72 (34–100)	74 (48–88)	74 (57–94)
Male	25 (69%)	99 (62%)	21 (75%)	20 (63%)
Female	11 (31%)	60 (38%)	7 (25%)	12 (37%)
Median hemoglobin (g/dl)	9.1	9.4	10.1	10.9
Median platelets (G/L)	65	90	92	75
Median ANC	1.32	1.42	7.87	5.67
Median monocyte count	0.22	0.24	1.96	2.58
Median BM Blast %	4	4	4	4
*IPSS*				
Low	5 (14%)	40 (25%)	12 (43%)	15 (47%)
Intermediate 1	17 (47%)	50 (31%)	10 (36%)	13 (41%)
Intermediate 2	3 (8%)	37 (23%)	3 (11%)	4 (12%)
High	9 (25%)	32 (20%)	3 (11%)	0 (0%)
# of mutations (median)	2*	1	3	2
*ASXL1* Mutation^*a*^				
*ASXL1* c.1934dupG	7 (19%)		8 (29%)	
Frameshift	25 (69%)		18 (64%)	
Nonsense	11 (31%)		10 (36%)	
HMA treatment	25 (69%)	89 (56%)	16 (57%)	15 (47%)
Allo-HSCT	7 (19%)	23 (15%)	3 (11%)	4 (13%)
Median OS (months)	15.5	17.9	11.9**	NR

*MDS* myelodysplastic syndrome, *CMML* chronic myelomonocytic leukemia, *MT* mutant, *WT*, wild type, *ANC* absolute neutrophil count, *BM* bone marrow, *IPSS* international prognostic scoring system, *HMA* hypomethylating agent, *allo-HSCT* allogeneic hematopoetic stem cell transplantation, *OS* overall survival *P < 0.0001; **P = 0.02

^a^ *ASXL1* missense mutations occurred in 29% (*n* = 15) and 7% (*n* = 2) of the MDS and CMML cohorts, respectively

Overall, *ASXL1* mutation was identified in 26% (*n* = 51) and 50% (*n* = 30) of MDS and CMML patients, respectively (*P* = 0.0008). Although the distribution of FS and nonsense (NS) mutations were similar between the MDS and CMML cohorts (Table 1), missense mutations represented only 7% (*n* = 2) of *ASXL1* mutations in the CMML cohort compared to 29% (*n* = 15) in the MDS cohort (*P* = 0.02). In patients with *ASXL1* FS and NS mutations, there were no significant differences compared to wildtype patients in the CMML or MDS subgroups with regards to cytopenias, monocyte count, bone marrow blasts or IPSS risk classification (Table 1). However, MDS patients with *ASXL1* mutations had a significant increased absolute number of gene mutations (median 2 versus 1, *P* < 0.0001), which approached significance in the CMML cohort (median 3 versus 2, *P* = 0.08). In regards to therapeutic intervention, there was no difference in utilization of hypomethylating agent therapy or allogeneic hematopoietic cell transplantation.

**Fig. 1:**
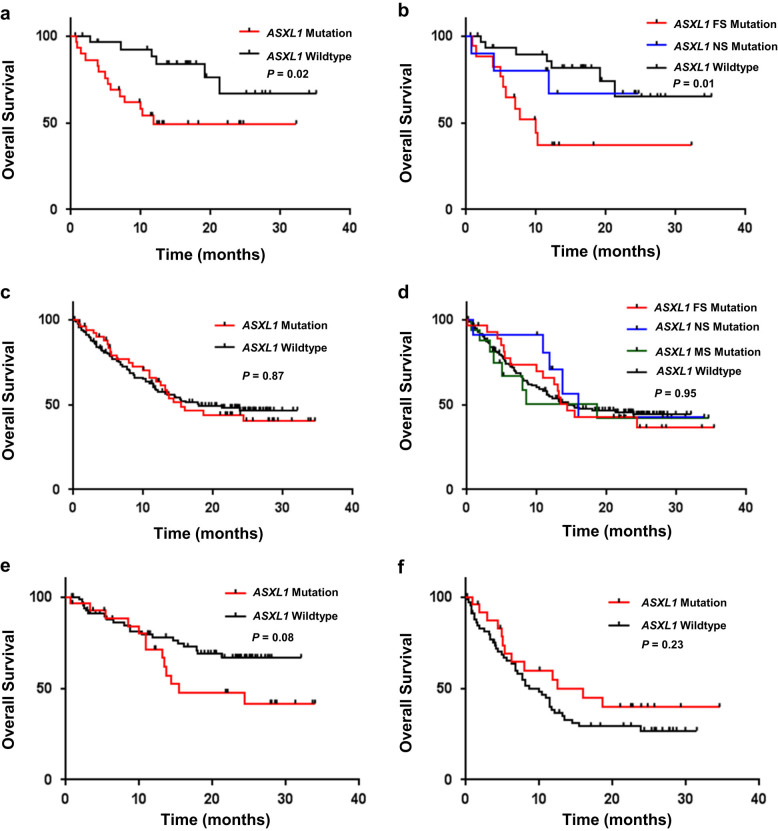
Overall survival by *ASXL1* mutation status and type of *ASXL1* mutation OS of CMML patients stratified by **a** *ASXL1* mutation status and **b** type of *ASXL1* mutation. OS of MDS patients stratified by **c** *ASXL1* mutation status and **d** type of *ASXL1* mutation. OS of *ASXL1* mutant and wildtype MDS patients with **e** low bone marrow blasts (<5%) and **f** excess blasts (≥5%). *FS* frameshift, *NS* nonsense, *MS* missense

We first evaluated the impact of *ASXL1* mutation on survival in the CMML cohort given prior data that found mutation to be predictive of inferior OS, albeit only when FS and NS mutations were included^4^. Indeed, CMML patients with *ASXL1* FS or NS mutations had inferior OS with a median OS of 11.9 months versus NR in WT patients (Fig. 1a, HR 2.97, 95% CI 1.21–7.06; *P* = 0.02). Of interest, type of mutation was predictive in the CMML cohort with a median OS of 9.9 months in *ASXL1* FS mutant patients versus not reached (NR) in NS or wildtype patients (Fig. 1b, *P* = 0.01). In comparison to WT patients, *ASXL1* FS patients had significantly inferior OS (median OS 9.9 months vs NR; *P* = 0.0026) while NS mutant patients had no difference in survival (*P* = 0.70). In multivariable analysis incorporating age, sex, and IPSS, *ASXL1* FS mutation had the greatest impact on OS (HR 5.87, 95% CI 1.98–17.4, *P* = 0.001). The clonal burden as defined by variant allele frequency of *ASXL1* FS or NS mutations did not further stratify survival in the CMML cohort. In contrast to the CMML cohort, *ASXL1* mutation had no impact on outcome in the MDS cohort with a median OS of 15.5 months versus 17.9 months in wildtype patients (Fig. 1c, HR 1.04, *P* = 0.87). Furthermore, type of mutation had no effect on survival with a median OS of 14.3 months with FS, 16.0 months with NS and 18.6 months with missense mutations (Fig. 1d, *P* = 0.95). Exclusion of *ASXL1* missense mutations had no impact on the prognostic impact of *ASXL1* mutations (median OS of 14.3 months FS/NS patients versus NR in wildtype patients; HR 1.18, *P* = 0.42). Subgroup analysis of *ASXL1* mutant MDS patients without excess blasts (i.e., <5%) identified a trend for inferior OS in the mutant cohort (median OS 15.5 months versus NR in the wildtype cohort; Fig. 1e, HR 1.85; *P* = 0.08). However, type of *ASXL1* mutation (*P* = 0.14) or restriction of missense mutations (*P* = 0.23) did not further stratify outcomes in this patient population. In contrast in patients with excess blasts, median OS was 12.5 months in *ASXL1* mutant patients versus 8.7 months in wildtype patients although not statistically significant (Fig. 1f, HR 0.68; *P* = 0.23).

We next evaluated differences between the molecular architecture of *ASXL1* mutant cases. CMML patients with *ASXL1* mutation had significantly increased co-occurrence of *SRSF2* (15.7% versus 5%, *P* = 0.01) as well as higher risk mutations including *RUNX1* (9.8% versus 3.6%; *P* = 0.04), *CBL* (7.8% versus 1.2%, *P* = 0.02) and *EZH2* (9.8% versus 0.8%, *P* = 0.0001). In addition, *ASXL1*/*TET2* co-mutation was significantly more common in CMML patients (30.8% versus 7.2%, *P* = 0.0001) with no statistically significant co-mutation in the MDS subgroup.

*ASXL1* mutations in myeloid malignancies have been shown to be loss-of-function mutations resulting in myeloid transformation via loss of polycomb repressive complex 2 (PRC2)-mediated H3K27 tri-methylation^10^. Furthermore, leukemogenesis in ASXL1-deficient cells occurs through cooperation with secondary mutations. Additionally, knockdown of *ASXL1* impairs granulomonocytic differentiation and leads to overexpression of PRC2 targets^11^. Patnaik and colleagues have evaluated the prognostic interplay of *ASXL1*/*TET2* mutation status in CMML and identified *TET2* mutant patients without *ASXL1* mutations to have improved OS in comparison to co-mutant patients which had the shortest survival^12^. Perhaps, critical to the prognostic relevance of *ASXL1* mutation is its collaboration with other mutations. To this regard, we highlight a significant correlation of higher risk mutations as well as the *ASXL1*/*TET2* co-mutant genotype in CMML compared to MDS patients.

Recent serial molecular annotation of *ASXL1* mutant MDS patients showed that the *ASXL1* clone was unchanged in all cases at the time of disease progression (*n* = 32) and was only predictive of survival when analysis was restricted to lower risk patients^13^. Our data support these findings as there was a trend for inferior OS in MDS patients without excess blasts. Nazha and colleagues recently created and validated a new molecular prognostic model by incorporating molecular data with IPSS-R risk categorization on a total of 429 MDS and 79 CMML patients where *ASXL1* mutation was not predictive of OS^14^. Altogether, these data suggest that although *ASXL1* mutations play a key role in the pathogenesis of MDS, they do not have prognostic relevance in higher risk populations. In contrast, our data further validate the prognostic role of *ASXL1* mutations in CMML. Although previous studies confirmed that missense mutations do not impact outcomes in CMML, our study suggests that FS mutations are the most prognostically relevant. Understanding the functional consequence on protein function based on type of *ASXL1* mutation needs to be evaluated in future study.

In conclusion, we have identified that only *ASXL1* FS mutations predict inferior survival in CMML while *ASXL1* mutation status was not predictive of inferior outcomes in the total cohort of MDS patients, regardless of type of mutation. Notably, the differential prognostic impact of *ASXL1* mutations in CMML versus MDS patients could be dependent on disease-specific secondary mutations that co-occur with *ASXL1* mutations. Furthermore, prognostic relevance of *ASXL1* mutations in MDS patients appears to be restricted to lower risk disease. Together, this study highlights significant heterogeneity in the prognostic relevance of *ASXL1* mutations in MDS and CMML.
